# External validation of a machine learning-based classification algorithm for ambulatory heart rhythm diagnostics in pericardioversion atrial fibrillation patients using smartphone photoplethysmography: the SMARTBEATS-ALGO study

**DOI:** 10.1093/europace/euaf031

**Published:** 2025-02-17

**Authors:** Jonatan Fernstad, Emma Svennberg, Peter Åberg, Katrin Kemp Gudmundsdottir, Anders Jansson, Johan Engdahl

**Affiliations:** Karolinska Institutet, Department of Clinical Sciences, Danderyd University Hospital, Entrévägen 2, 182 88 Stockholm, Sweden; Department of Cardiology, Danderyd University Hospital, Entrévägen 2, 182 88 Stockholm, Sweden; Karolinska Institutet, Department of Medicine, Huddinge, Karolinska University Hospital, Stockholm, Sweden; Karolinska Institutet, Department of Clinical Sciences, Danderyd University Hospital, Entrévägen 2, 182 88 Stockholm, Sweden; Karolinska Institutet, Department of Clinical Sciences, Danderyd University Hospital, Entrévägen 2, 182 88 Stockholm, Sweden; Department of Clinical Physiology, Danderyd University Hospital, Stockholm, Sweden; Karolinska Institutet, Department of Clinical Sciences, Danderyd University Hospital, Entrévägen 2, 182 88 Stockholm, Sweden; Department of Cardiology, Danderyd University Hospital, Entrévägen 2, 182 88 Stockholm, Sweden

**Keywords:** Atrial fibrillation, Atrial flutter, Photoplethysmography, Smartphone, Telemonitoring, Machine learning

## Abstract

**Aims:**

The aim of this study was to perform an external validation of an automatic machine learning (ML) algorithm for heart rhythm diagnostics using smartphone photoplethysmography (PPG) recorded by patients with atrial fibrillation (AF) and atrial flutter (AFL) pericardioversion in an unsupervised ambulatory setting.

**Methods and results:**

Patients undergoing cardioversion for AF or AFL performed 1-min heart rhythm recordings pericardioversion at least twice daily for 4–6 weeks, using an iPhone 7 smartphone running a PPG application (CORAI Heart Monitor) simultaneously with a single-lead electrocardiogram (ECG) recording (KardiaMobile). The algorithm uses support vector machines to classify heart rhythm from smartphone-PPG. The algorithm was trained on PPG recordings made by patients in a separate cardioversion cohort. Photoplethysmography recordings in the external validation cohort were analysed by the algorithm. Diagnostic performance was calculated by comparing the heart rhythm classification output to the diagnosis from the simultaneous ECG recordings (gold standard). In total, 460 patients performed 34 097 simultaneous PPG and ECG recordings, divided into 180 patients with 16 092 recordings in the training cohort and 280 patients with 18 005 recordings in the external validation cohort. Algorithmic classification of the PPG recordings in the external validation cohort diagnosed AF with sensitivity, specificity, and accuracy of 99.7%, 99.7% and 99.7%, respectively, and AF/AFL with sensitivity, specificity, and accuracy of 99.3%, 99.1% and 99.2%, respectively.

**Conclusion:**

A machine learning-based algorithm demonstrated excellent performance in diagnosing atrial fibrillation and atrial flutter from smartphone-PPG recordings in an unsupervised ambulatory setting, minimizing the need for manual review and ECG verification, in elderly cardioversion populations.

**Clinical Trial Registration:**

Clinicaltrials.gov, NCT04300270

## Graphical Abstract

**Graphical Abstract:**
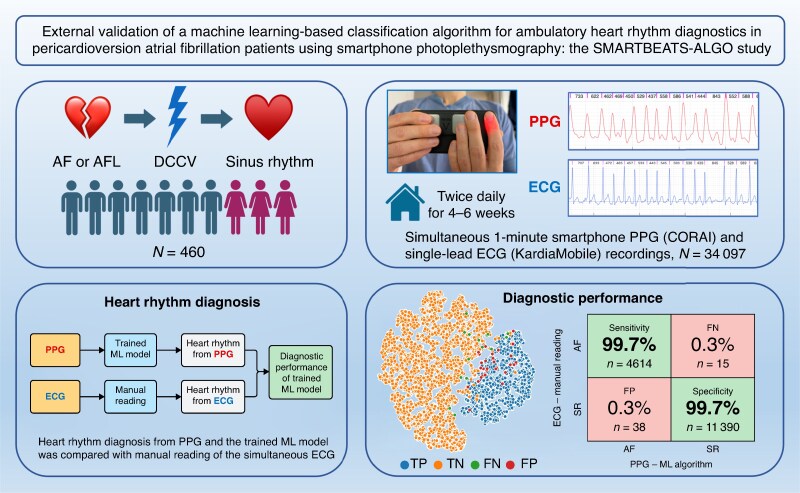

**What’s New?**

- In this large validation study of smartphone-based photoplethysmography (PPG) in the pericardioversion setting, the heart rhythm diagnosis of atrial fibrillation from an automatic machine learning (ML) algorithm showed an accuracy of 99.7% compared with manually interpreted simultaneous electrocardiograms.
- Inclusion of atrial flutter recordings into smartphone-PPG monitoring only marginally reduced the accuracy of the ML algorithm.
- Despite recorded in an unsupervised ambulatory setting, the ML algorithm demonstrated excellent diagnostic performance and the proportion of smartphone-PPG recordings having sufficient quality for diagnosis was high.

## Introduction

Atrial fibrillation (AF) has a global prevalence of 60 million,^1^ and increases the risk for complications such as all-cause death, ischaemic stroke, heart failure, and dementia.^2–5^ Atrial flutter (AFL) is a less prevalent cardiac arrhythmia, with similar complications and symptoms as AF.^5^

The European Society of Cardiology (ESC) guideline for the management of AF recommend a 12-lead electrocardiogram (ECG) (12L-ECG) recording, or a multiple- or single-lead ECG (1L-ECG) tracing, analysed manually by a physician for AF diagnosis.^5,6^ Smartphone photoplethysmography (PPG) can be used for management of AF/AFL.^5,6^ However, different smartphone-PPG methods are not equivalent; key differences include aspects such as measurement technology, ease of use, resulting signal quality, and diagnostic performance.^7–10^ Questions have been raised about the generalizability of the results of previous validation studies on various smartphone-PPG methods.^9^ Therefore, the current ESC and American Heart Association guidelines do not recommend smartphone-PPG for diagnosis of AF, without a confirmatory ECG recording.^5,6,11^

Most previous validation studies were conducted in a supervised healthcare setting, and even though measurements were made under direct supervision by healthcare personnel the quality of the PPG recordings was often insufficient for diagnosis.^12,13^ We addressed these concerns in a previous study where we investigated the diagnostic performance of manual reading of smartphone-PPG recordings.^7^

The use of diagnostic automatic algorithms based on artificial intelligence and machine learning (ML) within medicine, and cardiology, is increasing.^14^ Arrhythmia investigations, and opportunistic and systematic screening, for detection of AF/AFL are examples of clinical applications that generate large numbers of heart rhythm recordings where smartphone PPG could be useful. Accurate ML-based automatic heart rhythm classification algorithms have the potential to facilitate such clinical applications, by offloading the need for manual review of the generated smartphone-PPG recordings.

This study was designed to investigate the diagnostic performance of an automatic ML-based algorithm for heart rhythm diagnostics using smartphone-PPG recorded in an ambulatory real-world situation, compared with simultaneous 1L-ECG recordings, in patients with AF and AFL pericardioversion.

## Methods

### Study design and participants

In this prospective validation study, participants were included at the Department of Cardiology, Danderyd University Hospital, Stockholm, Sweden. Adult patients undergoing direct current cardioversion (DCCV) for persistent or recent-onset AF or AFL were included. Patients with a cardiac implantable electronic device were excluded.

### Smartphone-PPG device

Photoplethysmography is an optical method measuring variations in subcutaneous blood volume.^6^ The CORAI Heart Monitor PPG software application (Corai Medicinteknik AB, Stockholm, Sweden) is a Class IIb medical device running on the operating system of smartphones, such as the iOS (Apple Inc., Cupertino, CA, USA) and Android (Alphabet Inc., Mountain View, CA, USA) operating systems. CORAI uses the built-in sensors of the smartphone to record a high-resolution PPG measurement from the user’s fingertip positioned over the camera sensor.^7^

**Figure 1:**
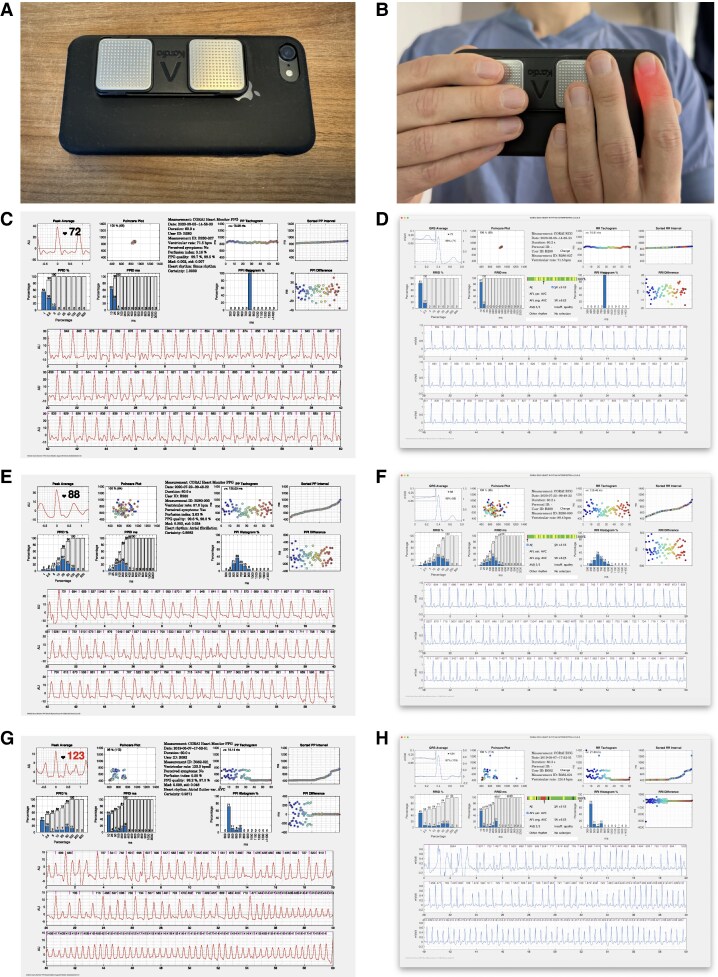
Simultaneous smartphone-PPG and single-lead ECG recordings with CORAI Heart Monitor and KardiaMobile. (*A*) The single-lead ECG device KardiaMobile was attached to the back of the smartphone case on an iPhone 7. (*B*) Shows the user handling and finger placements when performing a simultaneous smartphone-PPG recording with the CORAI Heart Monitor and single-lead ECG recording. The study participants performed both type of recordings simultaneously by placing a fingertip over the camera lens of the smartphone, at the same time as placing other fingers from both hands on the electrodes of the single-lead ECG device. (*C*, *E*, *G*) The CORAI PPG Report showing smartphone-PPG recordings with automatic heart rhythm diagnosis from the machine learning algorithm, it’s estimated degree of certainty of the diagnosis and the calculated signal quality of the PPG tracing. (*D*, *F*, *H*) The user interface in the software application and ECG reports used during manual reading for the single-lead ECG recordings. The reader would select the heart rhythm diagnosis for the shown ECG recording. For the recordings in (*C* and *D*) the heart rhythm was sinus rhythm, in (*E* and *F*) it was atrial fibrillation, and in (*G* and *H*) the heart rhythm was atrial flutter. AF, atrial fibrillation; AFL reg. AVC, atrial flutter with regular AV conduction; AFL var. AVC, atrial flutter with variable AV conduction; AVB, atrioventricular block; ECG, electrocardiogram; ES, extrasystolic beats; PPG, photoplethysmogram; SR, sinus rhythm.

The resulting PPG recording contains information on heart rhythm and heart rate.^6^ For each recording, a PPG report with tracings is generated by the CORAI application, allowing for a heart rhythm diagnosis to be determined through manual reading.^7^ The CORAI application can automatically decide on a heart rhythm diagnosis based on analysis of the PPG recording by a ML-based heart rhythm classification algorithm (*Figure 1*).

### Single-lead ECG device

KardiaMobile (AliveCor Inc., Mountain View, CA, USA) is a portable device that records a 1L-ECG and requires the presence of a smartphone that receives and processes the ECG during recording. KardiaMobile has been validated for the detection of several arrhythmias including AF and AFL.^15–17^ For each 1L-ECG recording in this study, an ECG report is generated, from which a heart rhythm diagnosis can be decided on by manual reading (*Figure 1*).

### Simultaneous PPG and ECG recordings

The study participants were provided with an iPhone 7 (Apple Inc., Cupertino, CA, USA) smartphone to use for the study duration. The 1L-ECG device was attached to the back of the smartphone’s case. For each recorded smartphone PPG, a simultaneous 1L-ECG recording was made; see *Figure 1*. The study participants were given automatic real-time feedback on the user handling by the CORAI application during recordings in the form of voice and text prompts to help improve signal quality for both PPG and ECG recordings. An update to the algorithms used in the automatic real-time user feedback system to analyse PPG signal quality in real-time was made in the second half of the participant inclusion in the external validation cohort and was available for all participants in the training cohort. The contents of the user feedback given remained the same after the revision.

### Heart rhythm recordings

#### Training cohort

In the training cohort, patients were screened for participation via telephone call 1–2 weeks prior to their scheduled DCCV treatment and were asked to attend a screening visit the following day. Included patients were instructed to record simultaneous 1-min PPG and 1L-ECG measurements at least twice daily until their scheduled DCCV. If the DCCV was successful, the patients were asked to continue recording at least twice daily for an additional 30 days following DCCV. The first recording was performed under supervision during the inclusion appointment.

#### External validation cohort

In the external validation cohort, patients eligible for DCCV were screened for participation on the day of treatment. After confirmation of AF or AFL on 12-lead ECG, and if no contraindications for DCCV were identified, patients were informed and asked about participation. Included patients were instructed to record simultaneous 1-min PPG and 1L-ECG measurements at least twice daily for 30 days following DCCV. The first simultaneous PPG and 1L-ECG recordings were made before the DCCV treatment. Patients not converted to sinus rhythm (SR) after DCCV, as recorded by 12-lead ECG, were excluded.

The recordings in both cohorts were manually monitored daily by the investigators. The ambulatory recordings were made unsupervised, i.e. without any assistance in device handling from the investigators. Patients with recurring recordings with heart rate lower than 40 b.p.m. or over 140 b.p.m. had a telephone consultation with a physician.

### Manual reading of ECG recordings

The manual reading of 1L-ECG recordings used a customized software platform; see *Figure 1*. The set of heart rhythm diagnoses available to the readers is reported in Supplementary material online, *Table S1*. A minimum of 30 s of each 1-min recording was required to have sufficient signal quality to qualify for a heart rhythm diagnosis.

The 1L-ECG recordings were considered the gold standard and were independently analysed by two cardiology consultants (J.E. and K.K.G.) with experience in 1L-ECG reading. In the case of disagreement, a third cardiologist (E.S.) analysed the recording blinded to the heart rhythm diagnoses selected by the two initial readers. If all three readers provided different diagnoses, the heart rhythm diagnosis was determined by consensus in a meeting. The 1L-ECG recordings for each pseudonymized patient were grouped together and presented chronologically.

### Diagnosis from automatic algorithm

The automatic ML-based algorithm uses support vector machines (SVM) to classify the heart rhythm from smartphone-PPG recordings. Support vector machines is a supervised learning model that maximizes the margin between the training patterns and the decision boundary, which are made up of the ‘support vectors’.^18^ Once a SVM model has been trained from labelled input, it can be used as a classifier for new, unseen, unlabelled data.

For the internal validation, the SVM classifier evaluated here was trained on PPG recordings made by patients in the separate training cohort, ensuring complete independence between internal and external validation data sets. The heart rhythm for the PPG recordings in the training cohort was decided on by manual reading. During the internal validation, the SVM classifier was trained using Leave-One-Subject-Out cross-validation (LOSO-CV).^19^ The final SVM model was trained with the PPG recordings from all the subjects in the training cohort and without any optimization of the hyperparameters.

**Figure 2:**
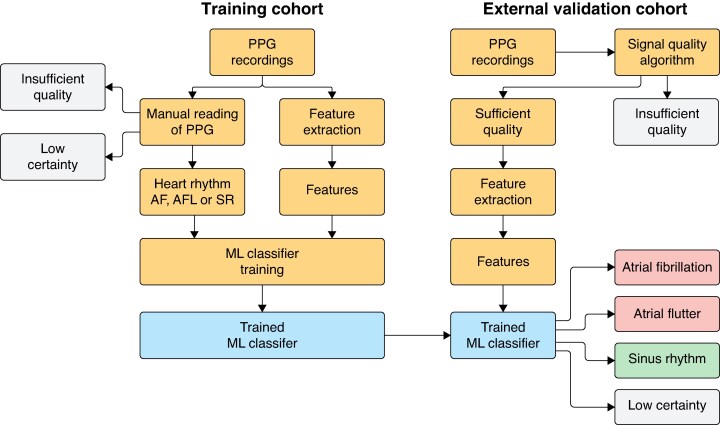
Training and external validation of the machine learning algorithm. The automatic machine learning algorithm was trained on PPG recordings in the separate training cohort. Heart rhythm determined by manual reading of the PPG recordings was used to train a support vector machine (SVM) classifier. The diagnostic performance of the trained SVM classifier was evaluated with PPG recordings in the external validation cohort compared with the heart rhythm from the manually interpreted simultaneous single-lead ECG recordings. AF, atrial fibrillation; AFL, atrial flutter; ML, machine learning; PPG, photoplethysmogram; SR, sinus rhythm.

The PPG recordings in the external validation cohort were analysed by the trained automatic algorithm (*Figure 2*), and diagnostic performance was calculated by comparing the heart rhythm classification output from the SVM classifier with the heart rhythm diagnosis from the simultaneous and manually read ECG recordings (gold standard). The signal quality of each PPG recording was determined by an automatic signal quality algorithm.

Besides classifying the heart rhythm, the trained SVM model outputs a parameter indicating the level of certainty of the made heart rhythm classification. The certainty parameter can be used to minimize the number of false positive and false negative heart rhythm classifications, by not accepting any heart rhythm classification output having low algorithmic classification certainty. A certainty score of 1.0 indicates maximum certainty in the classification output, while a score of 0.5 represents the lowest possible level of certainty for a binary classifier. Recordings with a classification certainty score < 0.9 were considered as having low certainty.

Recordings lacking a heart rhythm diagnosis on either ECG or PPG, due to insufficient signal quality, low algorithmic classification certainty, or for other reasons, were removed in the diagnostic performance calculations (see Supplementary material online, *Figure S1*).

### Statistics

Continuous variables, if normally distributed, were reported as mean with standard deviations, otherwise as median and interquartile ranges (IQRs) and dichotomous variables as proportions. The one-sample Kolmogorov–Smirnov test was used to test for normality. Diagnostic performance in terms of sensitivity, specificity, accuracy, and positive and negative predictive value (PPV and NPV) was calculated using 2 × 2 tables. Diagnostic performance was calculated separately including and excluding recordings read as AFL on ECG. When calculating the diagnostic performance including AFL recordings, AF and AFL were grouped together.^15,20,21^

Statistical significance for the diagnostic performance between subgroups of the study participants was calculated using a two-sided *χ*^2^ test. A *P*-value of < 0.05 was considered significant. The MATLAB software (The Mathworks Inc., Natick, MA, USA) was used for the statistical calculations.

### Ethics

The study was conducted according to the Declaration of Helsinki and was approved by the Regional Ethical Review Board, ref. no: 2018/1354–32, by the Swedish Ethical Review Authority, ref. no: 2019–02150 and 2022-02837, and by the Swedish Medical Products Agency, ref. no: 5.1-2022-26845. All participants signed informed consent.

## Results

### Study population

A total of 460 patients were included and the participants performed 34 097 simultaneous PPG and ECG recordings.

#### Training cohort

In the training cohort, 180 patients were included between September 2022 and June 2024 and recorded a total of 16 092 simultaneous PPG and ECG recordings, resulting in an average of 89.4 simultaneous recordings per patient. The median age in this cohort was 69.6 years and 27% (48/180) of the participants were female. The mean CHA_2_DS_2_-VA score was 2.0 (median 2; IQR 2) and the most reported EHRA score was III (41%). The main SVM classifier (‘the AF classifier’) was trained on data from 153 patients from the training cohort, with 11 749 PPG recordings, of which 5876 (50.0%) were labelled as SR and 5873 (50.0%) as AF. A second SVM model, that also classifies AFL recordings (‘the AFL classifier’), was trained on data from 180 patients from the training cohort, with 15 444 PPG recordings, of which 7895 (51.1%) were labelled as SR, 6998 (45.3%) as AF, and 551 (3.6%) as AFL. The full baseline characteristics of the training cohort are shown in *Table 1*. For further details of the internal validation, see Supplementary material online, *Figure S2*.

**Table 1:** Baseline characteristics of the study population

Variable	External validation cohort	Training cohort
Participants, ***n***	280	180
Age, median (IQR), years	69.0 (13.6)	69.6 (13.6)
Age, range, years	32.3–89.5	30.3–88.9
Female, ***n*** (%)	86 (30.7)	48 (26.7)
BMI (kg/m^2^), median (IQR)	26.6 (5.1)	27.1 (5.5)
Pre-DCCV heart rhythm from 1L-ECG, ***n*** (%)	AF: 230 (82.1)	–
	AFL: 40 (14.3)	
	Insufficient quality: 10 (3.6)	
Hyperlipidaemia, ***n*** (%)	63 (22.5)	67 (37.2)
Hypertension, ***n*** (%)	164 (58.6)	102 (56.7)
CHF, ***n*** (%)	53 (18.9)	17 (9.4)
Diabetes, ***n*** (%)	32 (11.4)	19 (10.6)
Stroke or TIA, ***n*** (%)	27 (9.6)	18 (10.0)
Vascular disease, ***n*** (%)	32 (11.4)	23 (12.8)
CHA_2_DS_2_-VA score, mean, median, (IQR)	2.1, 2, (2)	2.0, 2, (2)
EHRA score, proportion	I: 4.0%	I: 7.2%
	IIa: 9.7%	IIa: 20.0%
	IIb: 33.1%	IIb: 30.6%
	III: 52.5%	III: 40.6%
	IV: 0.7%	IV: 1.7%
DCCV scheduled, ***n*** (%)	214 (76.4)	180 (100)
DCCV within 48 h of AF/AFL initiation, ***n*** (%)	66 (23.6)	0 (0)
First time DCCV, ***n*** (%)	132 (47.1)	130 (72.2)
Number of previous DCCV, mean	2.78	0.66
Number of previous DCCV, range	0–35	0–10
Previous ablation procedure, ***n*** (%)	26 (9.3)	9 (5.0)
OAC treatment, ***n*** (%)	265 (94.6)	180 (100)
Warfarin, ***n*** (%)	18 (6.4)	2 (1.1)
DOAC, ***n*** (%)	247 (88.2%)	178 (98.9)
Mobile phone ownership, ***n*** (%)	279 (99.6)	180 (100)
Non-smartphone, ***n*** (%)	23 (8.2)	1 (0.6)
Smartphone, ***n*** (%)	256 (91.4)	179 (99.4)
iOS, ***n*** (%)	167 (65.2)	126 (70.0)
Android, ***n*** (%)	88 (34.4)	153 (29.4)
Windows mobile, ***n*** (%)	1 (0.4)	0 (0)

AF, atrial fibrillation; AFL, atrial flutter; BMI, body mass index; BP, blood pressure; CHF, congestive heart failure; DCCV, direct current cardioversion; DOAC, direct oral anticoagulants; ECG, electrocardiogram; IQR, interquartile range; OAC, oral anticoagulation; TIA, transient ischaemic attack.

#### External validation cohort

**Figure 3:**
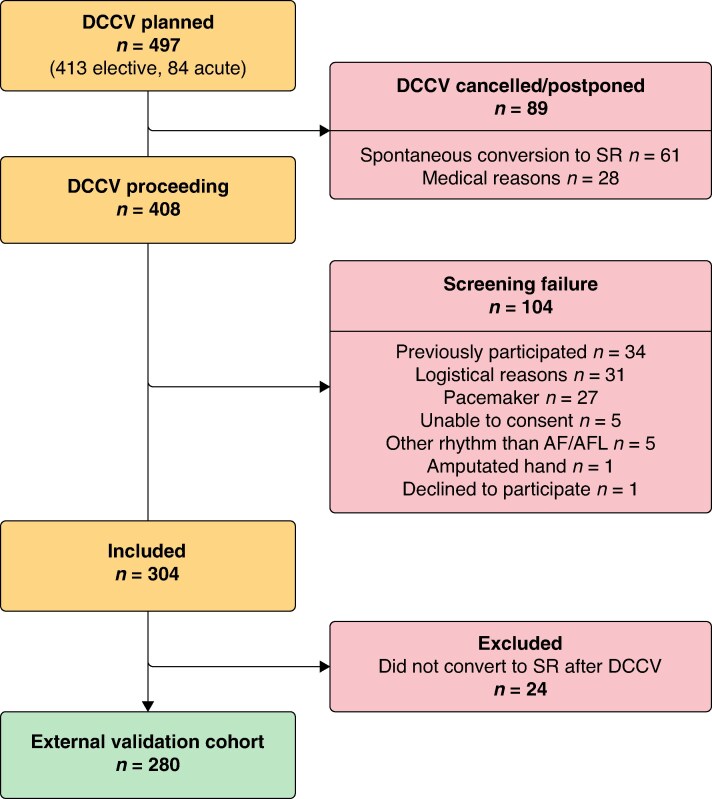
Study participant flow chart in the external validation cohort. The most common medical reason for DCCV cancellation was non-compliance to anticoagulation therapy. Logistical reasons for screening failure included insufficient availability of study devices, the patients having the DCCV procedure before they could be asked for participation, and inability to return the study device. AF, atrial fibrillation; AFL, atrial flutter; DCCV, direct current cardioversion; SR, sinus rhythm.

Between November 2018 and July 2020, 304 patients were included, of which 280 converted to SR after DCCV constituting the final external validation cohort (*Figure 3*). The median age in the external validation cohort was 69.0 years and 31% (86/280) of the participants were female. The mean CHA_2_DS_2_-VA score was 2.1 (median 2; IQR 2) and the most reported EHRA score was III (53%). Of the 280 participants in the cohort, 214 (76.4%) had an elective DCCV procedure, and for 66 (23.6%), the procedure was performed within 48 h of AF/AFL initiation. Pre-DCCV 1L-ECG recordings were interpreted as AF in 82.1% (230/280), as AFL in 14.3% (40/280), and as having insufficient quality for diagnosis in 3.6% (10/280) of the participants.^7^ The baseline characteristics of the external validation cohort are further described in *Table 1*.

The participants in the external validation cohort recorded a total of 18 005 simultaneous PPG and ECG recordings, with an average of 64.3 simultaneous recordings per patient.

In total, eight patients across both cohorts that had recurring recordings with a heart rate lower than 40 b.p.m. or over 140 b.p.m. had a telephone consultation with a physician.

### Overall diagnostic performance

**Figure 4:**
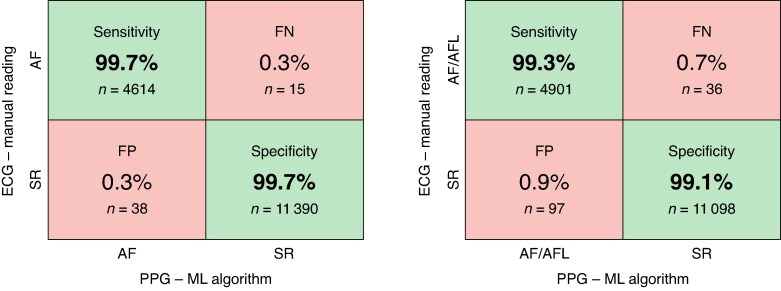
Overall diagnostic performance. This figure shows the diagnostic performance of the automatic machine learning algorithm that classifies the heart rhythm from smartphone-PPG recordings made with CORAI Heart Monitor compared with manual Reading of the simultaneous single-lead ECG recordings with KardiaMobile, with AFL recordings excluded (left) and with AFL recordings included (right). Example recordings of FP and FN classification outputs are available in Supplementary material online, *Figure S7*. AF, atrial fibrillation; AFL, atrial flutter; ECG, electrocardiogram; FN, false negatives; FP, false positives; ML, machine learning; PPG, photoplethysmogram; SR, sinus rhythm.

Algorithmic classification of the PPG recordings in the external validation cohort, excluding AFL recordings, diagnosed AF (sensitivity) in 99.7% [95% confidence interval (CI): 99.5–99.8%], and SR (specificity) in 99.7% (95% CI: 99.6–99.8%) of the recordings compared with manually interpreted ECG recordings, with an overall accuracy of 99.7% (95% CI: 99.6–99.8%) (*Figure 4*). Positive predictive value and NPV were 99.2% (95% CI: 98.9–99.4%) and 99.9% (95% CI: 99.8–99.9%), respectively. F1-score was 99.4% (95% CI: 99.2–99.6%) and the area under the ROC curve (AUROC) was 0.999.

Excluding ECG recordings with AFL, 93.9% (263/280) of the study participants had complete agreement between all ECG and PPG recordings if dichotomized into SR vs. AF, and 97.5% (273/280) of the study participants had at most one recording with disagreement (see Supplementary material online, *Figure S3*).

### Atrial flutter

Algorithmic classification using the AFL classifier, including recordings read as AFL on ECG, diagnosed AF/AFL (sensitivity) in 99.3% (95% CI: 99.0%–99.5%) and SR (specificity) in 99.1% (95% CI: 99.0–99.3%) of the recordings, with an overall accuracy of 99.2% (95% CI: 99.0–99.3%). Positive predictive value and NPV were 98.1% (95% CI: 97.7–98.4%) and 99.7% (95% CI: 99.6–99.8%), respectively (*Figure 4*). AUROC was 0.992 and F1-score was 98.7% (95% CI 98.%3–99.0%). More details of the external validation including AFL recordings are available in Supplementary material online, *Figure S4*.

### Manual reading of ECG recordings

Of the 1L-ECG recordings in the external validation cohort, 65.6% (11 820/18 005) were read as SR, 27.3% (4911/18 005) as AF, 2.0% (362/18 005) as AFL, and 4.9% (882/18 005) as having insufficient quality.

For 1.2% (223/18 005) of the 1L-ECG recordings, an inter-observer disagreement of the selected heart rhythm occurred (one of the ECG readers had selected SR, and the other reader had selected either AF or AFL). Of these inter-observer disagreements, the abnormal heart rhythm was AFL in 0.6% (107/18 005) and AF in 0.6% (116/18 005) of the 1L-ECG recordings. Data on manual reading of 1L-ECG recordings have been published in more detail previously.^7^

### Quality of PPG and ECG recordings

**Figure 5:**
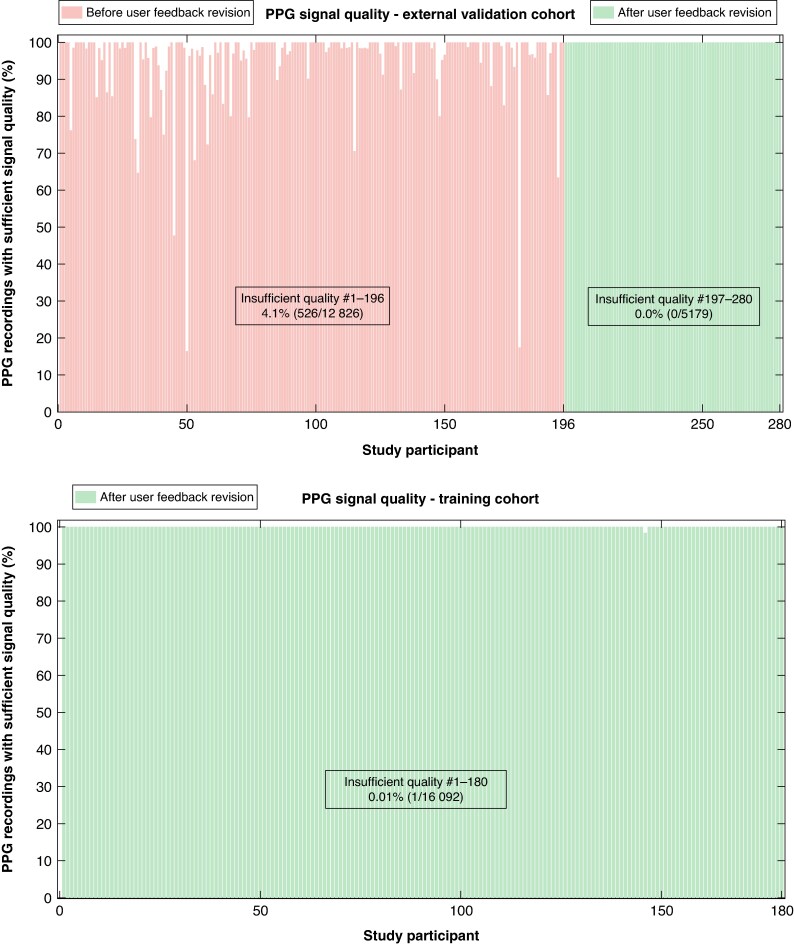
Signal quality of the smartphone-PPG recordings made in the external validation cohort (top) and in the training cohort (bottom) as determined by the automatic signal quality algorithm. This figure shows the signal quality of the smartphone-PPG recordings for each of the study participants. The proportion of smartphone-PPG recordings with sufficient quality to make a heart rhythm diagnosis, as decided by the automatic signal quality algorithm, are shown on the y-axis. The study participants were given automatic real-time feedback of the user handling by the CORAI Heart Monitor application during the recordings to help preserve the signal quality of the recordings. A revision to this automatic real-time feedback improved the signal quality of the smartphone-PPG recordings further substantially. The revised real-time feedback was available for the final 84 study participants, #197 to #280, in the external validation cohort, and for all the participants in the training cohort. PPG, photoplethysmogram.

Of all the PPG recordings in the external validation cohort, 2.9% (527/18 005) had insufficient signal quality for diagnosis based on automatic algorithm analysis of signal quality, compared with 4.9% (882/18 005) of the ECG recordings (*P* < 0.001). For details on ECG signal quality per study participant, see Supplementary material online, *Figure S5*. Following a revision to the automatic real-time user feedback given during PPG recording for preserving signal quality, PPG signal quality improved (*Figure 5*). Of note, none (0%, 0/5179) of the PPG recordings from the final 84 included patients in the external validation cohort had insufficient quality for diagnosis based on automatic algorithm analysis of signal quality, compared with 4.1% (526/12 826) for the participants (*n* = 196) included prior to the real-time user feedback update. For the PPG recordings in the training cohort, 0.01% (1/16 092) had insufficient quality for diagnosis based on automatic algorithm analysis of signal quality.

The diagnostic performance for the PPG recordings made by these final 84 participants in the validation cohort was significantly higher with regard to sensitivity, accuracy, and NPV compared with the participants (*n* = 196) included prior to the real-time user feedback update but was not significantly different for specificity and PPV (*Table 2*).

**Table 2:** Diagnostic performance of the machine learning algorithm before and after revisions to the automatic real-time user feedback that significantly increased the signal quality of the PPG recordings, with AFL recordings excluded

Diagnostic performance	Before revision	After revision	*P*
Participants, ***n***	196	84	–
Recordings, ***n***	12 826	5179	–
Recording duration	60 s	60 s	–
PPG recordings with insufficient quality, ***n*** (%)	526 (4.1)	0 (0)	<0.0001
PPG recordings with low certainty algorithmic classification, ***n*** (%)	164 (1.4)	29 (0.6)	<0.0001
Sensitivity (%)	99.6 [99.4–99.8]	100.0 [100.0–100.0]	0.0411
Specificity (%)	99.6 [99.5–99.7]	99.8 [99.6–99.9]	0.1170
Accuracy (%)	99.6 [99.5–99.7]	99.8 [99.7–99.9]	0.0195
PPV (%)	99.2 [98.9–99.5]	99.2 [98.7–99.8]	0.9137
NPV (%)	99.8 [99.7–99.9]	100.0 [100.0–100.0]	0.0065

Corresponding 95% confidence intervals are shown within brackets.

AFL, atrial flutter; NPV, negative predictive value; PPG, photoplethysmography; PPV, positive predictive value.

### Certainty of algorithmic heart rhythm classification

**Figure 6:**
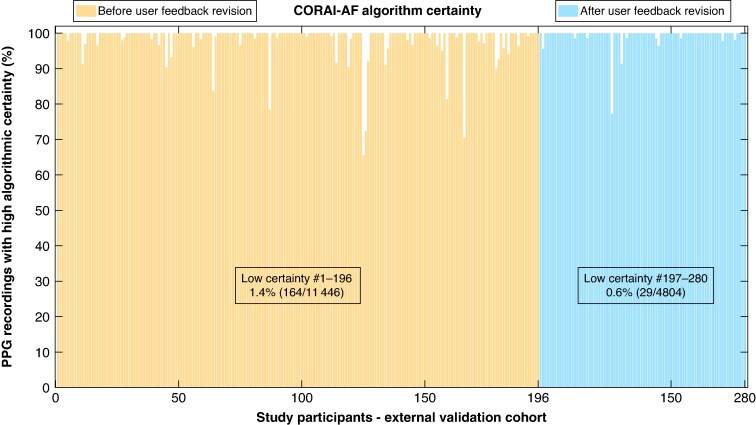
Algorithmic certainty of the made heart rhythm diagnosis for the PPG recordings in the external validation cohort, excluding AFL recordings, shown as the proportion having high diagnostic certainty for each study participant and compared before and after the update to the automatic real-time user feedback. PPG, photoplethysmogram.

Among the recordings in the external validation cohort that had sufficient quality on both ECG and PPG, and excluding AFL recordings, only 1.2% (193/16 250) had a low degree of certainty of the heart rhythm classification output from the AF classifier. After the update to the real-time user feedback this metric improved, to 0.6% (29/4804) for the final 84 compared with 1.4% (164/11 446) for the participants included prior (*n* = 196), see *Figure 6*. The combined proportion of PPG recordings excluded due to either low classification certainty or insufficient signal quality for those final 84 participants was only 0.6% (29/4804). When AFL recordings were included, 2.9% (475/16 607) of the PPG recordings in the external validation cohort had a low degree of certainty of the heart rhythm classification output from the AFL classifier. The degree of certainty for the PPG recordings in the external validation cohort can be seen in Supplementary material online, *Figure S6*.

**Figure 7:**
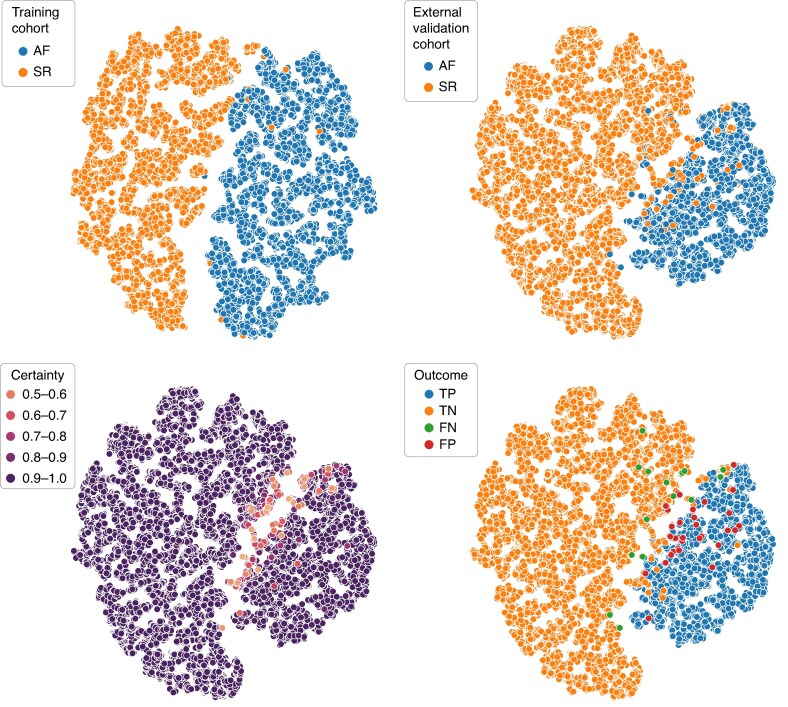
Visualization of the smartphone-PPG recordings included in the training cohort (top-left) and the external validation cohort (top-right), excluding AFL recordings, using a t-SNE algorithm that reduces each PPG recording to a single point in 2D space clustering similar PPG recordings together, showing separation between PPG recordings where the heart rhythm is SR and where it is AF. Diagnostic performance of the ML-based automatic algorithm trained on PPG recordings in the training cohort and externally validated on PPG recordings in the external validation cohort are visualized (bottom-right) and the prediction certainty of the recordings is shown (bottom-left). AF, atrial fibrillation; FN, false negatives; FP, false positives; ML, machine learning; PPG, photoplethysmogram; SR, sinus rhythm; TN, true negatives; TP, true positives.

The t-distributed stochastic neighbour embedding algorithm (t-SNE) is a dimensionality reduction technique that helps to grasp the structure and content of large data sets.^22^ When a t-SNE algorithm is used to visualize the PPG recordings in the external validation cohort, without any knowledge of the underlying heart rhythm, the algorithm is able to cluster SR and AF recordings in to two separate groups. The recordings with low classification certainty are then located near or on the border between the two groups, see *Figure 7*.

### Algorithmic diagnosis vs. manual reading

The diagnostic performance of the ML algorithm was compared with the previously reported diagnostic performance for manual reading of the PPG recordings in the external validation cohort, excluding AFL recordings.^7^ The results for the automatic algorithm were not significantly different for specificity or PPV but were significantly better for sensitivity, overall accuracy and NPV, see *Table 3*.

**Table 3:** Comparison between diagnostic performance of algorithmic classification and manual reading of heart rhythm from ambulatory smartphone-PPG recordings, excluding AFL recordings

Diagnostic performance	Manual reading^7^		ML algorithm	*P*
Participants, ***n***		280		–
Recordings, ***n***		18 005		–
PPG recordings with insufficient quality, ***n*** (%)	561 (3.1)		526 (2.9)	0.2801
Sensitivity (%)	99.0 [98.7–99.3]		99.7 [99.5–99.8]	<0.0001
Specificity (%)	99.7 [99.6–99.8]		99.7 [99.6–99.8]	0.7915
Accuracy (%)	99.5 [99.4–99.6]		99.7 [99.6–99.8]	0.0087
PPV (%)	99.2 [99.0–99.5]		99.2 [98.9–99.4]	0.8445
NPV (%)	99.6 [99.5–99.7]		99.9 [99.8–99.9]	<0.0001

AFL, atrial flutter; NPV, negative predictive value; PPG, photoplethysmography; PPV, positive predictive value.

## Discussion

In this study, we report excellent diagnostic performance for heart rhythm diagnostics by a ML-based automatic algorithm for smartphone PPG recordings as compared with 1L-ECG in an ambulatory elderly population pericardioversion. When AFL recordings were included the performance remained strong. Additionally, the classification certainty parameter could assist physicians estimate the accuracy of the algorithm’s diagnosis for individual recordings. Despite ambulatory recordings, the proportion of PPG recordings having sufficient signal quality for diagnosis was high.

Performing a validation study in an ambulatory setting can be challenging compared with in-office settings, since achieving high adherence may be difficult and supervision from healthcare professionals is not readily available.^23^ Even though most previous validation studies of smartphone PPG for AF detection were in supervised healthcare settings, the results in this unsupervised study show increased diagnostic performance in comparison.^9^

Due to its frequent presentation with regular rhythm and with a heart rate within the range of normal SR, AFL can sometimes be difficult to diagnose using 1L-ECG.^19,20^ For generalizability purposes, we chose to include patients with AFL in this study and although the overall accuracy remained good, the diagnostic performance decreased when AFL recordings were included.^9^ However, compared with manual reading, the ML-based algorithm showed better performance in diagnosing AFL.^7^

There is a relation between the signal quality of smartphone-PPG recordings and the diagnostic performance for AF diagnosis.^7^ Previous validation studies for smartphone-PPG methods reporting PPG signal quality had insufficient quality for diagnosis in 14–32% of the recordings.^12,13,24^ The proportion of PPG recordings having insufficient quality for diagnosis in the external validation cohort in this study was low (2.9%) in comparison. Using an automatic algorithm for real-time user feedback PPG signal quality can improve. In our study, all (100%, 5179/5179) of the PPG recordings performed by the final 84 included patients in the external validation cohort had sufficient quality for diagnosis, based on automatic algorithm analysis of signal quality. In the training cohort, 99.9% (16 091/16 092) of the PPG recordings had sufficient quality for diagnosis. The improvement in signal quality accompanied an improvement in diagnostic performance, underlining the importance of having an effective system incorporating real-time user feedback for this purpose. There was no difference between the proportions of PPG recordings having insufficient quality for diagnosis based on automatic algorithm analysis or based on manual reading, see *Table 3*.

A ML algorithm incorporating uncertainty estimation can help clinicians gauge how much they can trust its predictions, enabling more informed and confident decision-making in patient care.^25^ Many ML-based systems in recent medical literature overlook this aspect, though it has been argued that models should abstain from diagnosis when classification certainty falls below a set threshold to reduce false positives and negatives.^25,26^ In this study, we show that the performance of the algorithm increases further if PPGs with a low degree of classification certainty is removed, and we believe this could be a feature that has the potential to aid a new reader of PPGs.

In this study, we excluded PPG recordings that had a low degree of certainty for the heart rhythm classification output from the ML classifier. The proportion of PPG recordings having low classification certainty was small and improved significantly for the recordings made after the update to the real-time user feedback, showing that improved PPG signal quality also increased the certainty of the heart rhythm classifications (*Figure 6*).

The combined proportion of PPG recordings excluded for insufficient signal quality or low classification certainty remained low (4.0%, 719/18 005) compared to the proportions excluded by insufficient signal quality in previous validation studies for other smartphone-PPG methods^12,13,24^ and was only 0.6% (29/4804) for recordings made by the final 84 participants in the external validation cohort. Unsurprisingly, when AFL recordings were included the proportion of PPG recordings having low classification certainty increased. The high proportion of PPG recordings with sufficient quality for diagnosis in combination with an accurate ML-based heart rhythm classifier reporting classification certainty, helped to minimize the proportions of recordings with false positive and false negative results.

There are multiple clinical applications for heart rhythm diagnostics for which the use of smartphone-PPG recordings analysed automatically with an accurate ML-based diagnostic algorithm could be beneficial. These include opportunistic and systematic screening for AF in primary and secondary (post-stroke) prevention settings, as recommended by ESC guidelines.^5^

For both population-based and post-stroke screening, a longer ambulatory monitoring duration could be beneficial for AF detection.^27,28^ However, since the availability of ambulatory ECG-devices is a finite resource and often need to be returned for use by other people, the monitoring duration for ambulatory ECG-devices are usually limited to shorter durations. Since smartphones are ubiquitous and recordings with smartphone PPG can use the patient’s own device, the monitoring duration would not be restricted by the need to return a monitoring device. If smartphone PPG was used for systematic screening a physical visit would not be necessary to initiate the process, which would be beneficial for people living far away from the nearest healthcare centre or for people with limited mobility. However, participation rates have been low or unknown in reported population-based screening studies with digital and site-less designs.^29–31^

In the current guidelines, it is recommended that an AF diagnosis is confirmed by an additional recording using ECG when AF is detected from a smartphone-PPG recording.^5,11^ As the basis for this recommendation, the ESC guideline references a systematic review from 2022 that concludes that the validation studies of smartphone PPG for AF detection were deemed to be small, of low-quality, and to have a high risk of patient selection bias.^5^ Concerns on the generalizability of the results on previous validation studies have been made also due to the exclusion of high proportions of recordings from final analysis.^9^ Deciding heart rhythm from the majority result of multiple consecutive PPG recordings and presenting results for shorter duration recordings from selected high-signal-quality PPG fragments within longer duration recordings are other examples of how the methodology in previous studies has differed from real-world usage.^12,13,32,33^ A need for validation studies to be set in real-world-like scenarios has been identified.^9,13^

In this study, we show that, with real-time user feedback and a ML classification algorithm incorporating uncertainty estimation, smartphone PPG can achieve excellent diagnostic accuracy in an ambulatory elderly cardioversion population. If comparable diagnostic performance is confirmed in populations without a prior diagnosis, the need for ECG verification may be reduced for new AF diagnoses. Adherence to the smartphone-PPG method studied here and the observed diagnostic performance may vary across different populations, such as in AF screening among high-risk populations. Positive predictive value, in particular, may be reduced in populations with a lower prevalence of AF. In a study on app-based rhythm monitoring in AF patients, the youngest age tertile (<59 years) exhibited the lowest motivation and adherence to smartphone PPG, whereas the oldest age tertile (>68 years) showed the highest motivation and adherence.^34^ Further studies on a broader population, including people without an existing AF diagnosis, are warranted.

For patients already diagnosed with AF, smartphone-PPG recordings can safely be used to guide physicians in caring for AF patients for ambulatory rate and rhythm control, for example to detect relapse of and paroxysmal AF in pericardioversion and periablation settings.^6^

### Strengths and limitations

This study has several strengths, including investigating the diagnostic performance of a smartphone-PPG method in a real-world situation, using ambulatory unsupervised smartphone-PPG recordings, compared with simultaneous ECG recordings, investigating diagnostic performance using external validation with separate training and external validation cohorts, and including unselected DCCV patients with AFL as well as with AF.

The PPG recordings exhibited high signal quality, resulting in a large proportion of PPG recordings having sufficient quality for diagnosis which likely contributed to the robust diagnostic performance achieved. Using manual reading of PPG recordings in training the ML classifier helped minimize any reading errors occurring due to low signal quality, since almost all PPG recordings in the training cohort had sufficient quality for diagnosis.

In addition, smartphone-PPG recordings with low algorithmic classification certainty were excluded, helping to minimize the number of false positive and false negative heart rhythm diagnoses. The combined proportion of PPG recordings excluded due to either low classification certainty or insufficient signal quality remained small.

This study has several limitations, including using a 1L-ECG device as the reference method. The 1L-ECG has limitations compared with 12L-ECG for arrhythmia diagnosis.^35^ The 1L-ECG reference device has been validated against 12L-ECG for the diagnosis of AF.^16^

As simultaneous smartphone-PPG and 1L-ECG recordings were performed, the user experience and handling differed from the standard situation, where only one modality is recorded. This may have resulted in lower quality of both PPGs as well as 1L-ECGs compared with non-simultaneous recordings.

The study focused on an elderly population undergoing DCCV, thus AF/AFL patients deemed unsuitable for the procedure were not included. This may have introduced a selection bias, as DCCV patients are likely to be healthier given their eligibility for a rhythm control strategy. In addition, patients may be more motivated to participate, potentially influencing adherence to ambulatory heart rhythm recordings. Consequently, the proportions of normal and abnormal heart rhythm findings observed in this study may not reflect those in other patient groups or clinical scenarios.

Photoplethysmography recordings can be made also with devices using other form factors, such as smartwatches.^36^ However, the diagnostic performance of the studied ML classification algorithm for PPG recordings made with other types of devices than smartphones were not studied here.

This study has only investigated the diagnostic performance for recordings made with one smartphone-PPG system. Since different smartphone-PPG methods vary significantly in critical aspects such as measurement technology, resulting signal quality and diagnostic performance, the results and clinical applications suggested in this study are not generalizable to other smartphone-PPG systems.

## Conclusion

A machine learning-based algorithm demonstrated excellent performance in diagnosing atrial fibrillation and atrial flutter from smartphone-PPG recordings in an unsupervised ambulatory setting, minimizing the need for manual review and ECG verification, in elderly cardioversion populations.

## Supplementary Material

euaf031_Supplementary_Data

## Data Availability

The data underlying this article will be shared on reasonable request to the corresponding author.
