# Supplementary material for: External validation of a machine learning-based classification algorithm for ambulatory heart rhythm diagnostics in pericardioversion atrial fibrillation patients using smartphone photoplethysmography: the SMARTBEATS-ALGO study
Source: Europace. 2025 Feb 17;27(4):euaf031. doi: 10.1093/europace/euaf031 (PMC11965787; doi:10.1093/europace/euaf031)
Supplement: euaf031_Supplementary_Data [file euaf031_supplementary_data.pdf]

Supplementary data. https://doi.org/10.1093/europace/euaf031

Supplementary data

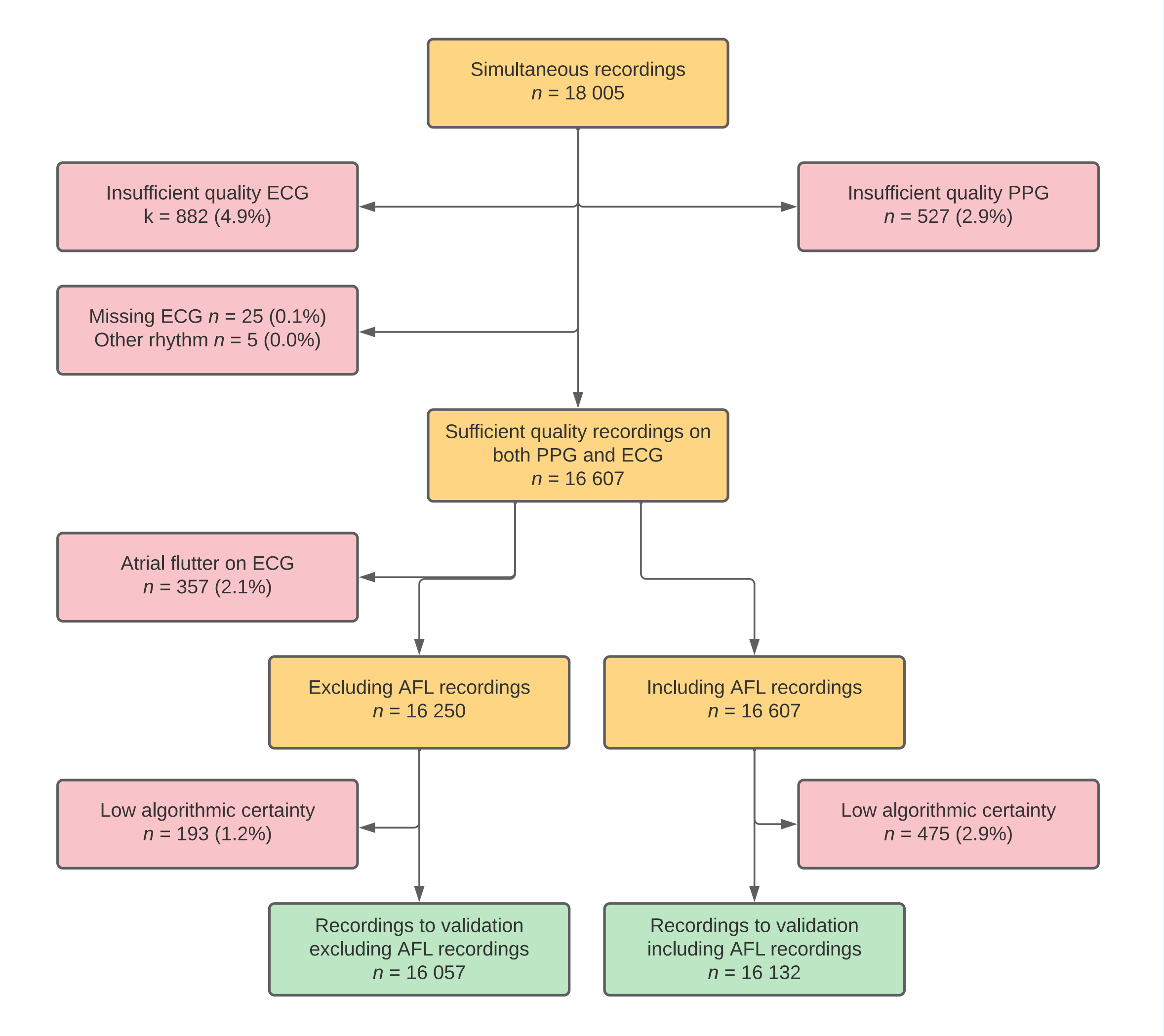

Supplementary figure S1 PPG and ECG recordings in the external validation cohort**.** To calculate diagnostic performance, heart rhythm diagnoses for both the smartphone-PPG recording and the simultaneous single-lead ECG recording must be present. Reasons for lack of diagnosis include missing recordings, insufficient quality, and other heart rhythms. "Other rhythm" was mostly selected for recordings having a heart rhythm change mid-recording. PPG recordings with low algorithmic classification certainty were excluded, with and without AFL recordings.
PPG: photoplethysmogram; ECG: electrocardiogram; AFL: atrial flutter;

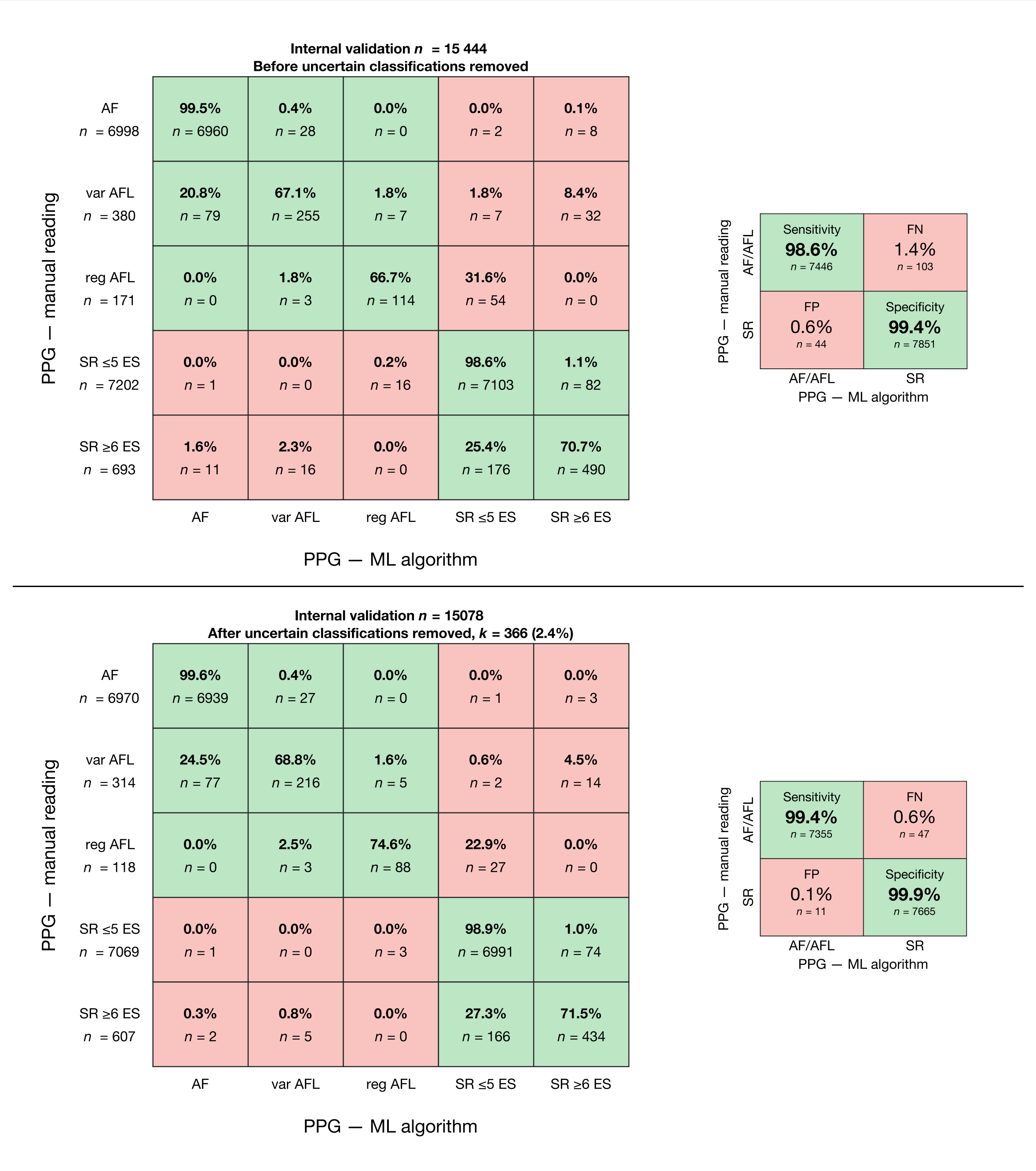

Supplementary figure S2 Confusion matrices for the internal validation, including AFL recordings, before **(top)** and after **(bottom)** removal of PPG recordings with a low degree of classification certainty. In the 2x2 matrices **(right)** AF and AFL recordings are grouped together. AF: atrial fibrillation; var AFL: atrial flutter with variable AV conduction; reg AFL: atrial flutter with regular AV conduction; SR: sinus rhythm; ES: extrasystolic beats; PPG: photoplethysmogram; ECG: electrocardiogram; ML: machine learning; FN: false negatives; FP: false positives;

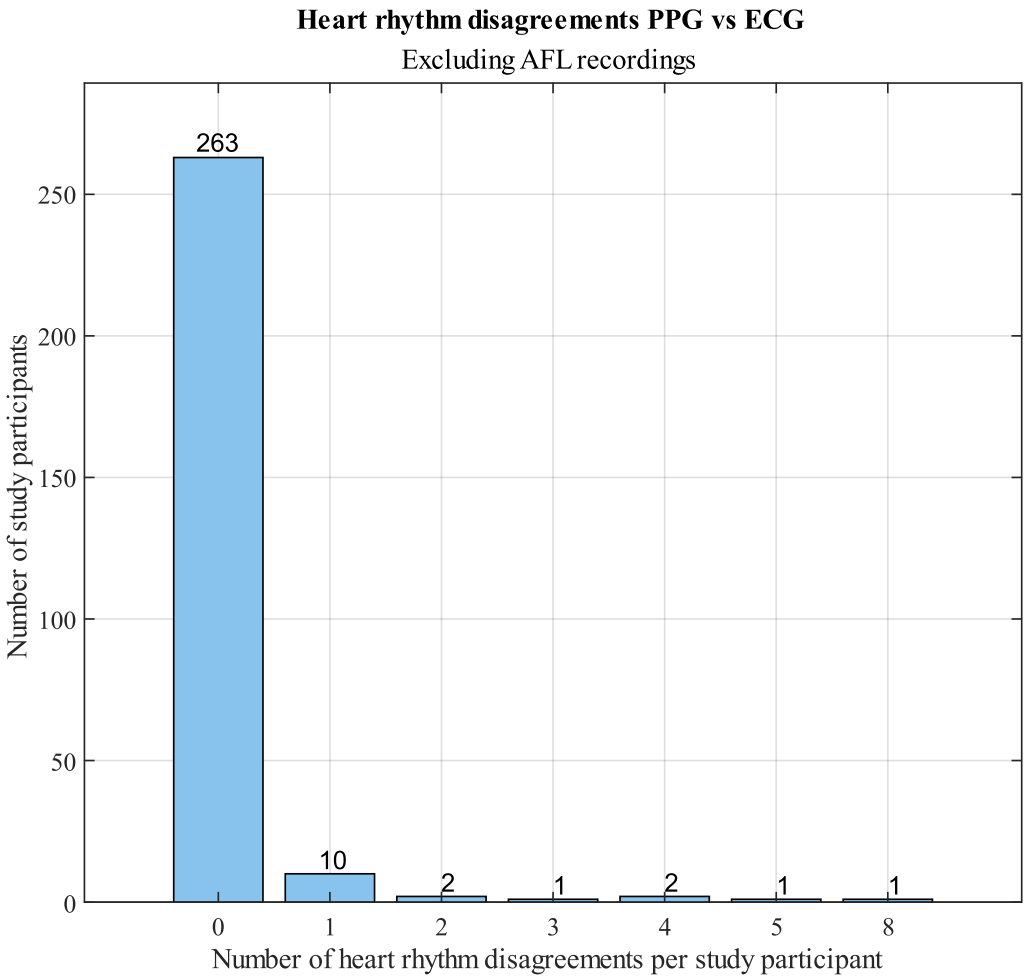

Supplementary figure S3 Diagnostic accuracy for a machine learning based heart rhythm classifier from smartphone-PPG recordings using CORAI Heart Monitor, per study participant in the external validation cohort, excluding AFL recordings. The number of study participants with the same number of disagreements in the heart rhythm between the machine learning algorithm diagnosis from smartphone-PPG, and from the manual reading of single-lead ECG are displayed together. Excluding AFL recordings, 263 out of the 280 (93.9%) study participants showed no disagreement at all, and 273 (97.5%) of the study participants had at most one disagreement.
AFL: atrial flutter; PPG: photoplethysmogram; ECG: electrocardiogram;

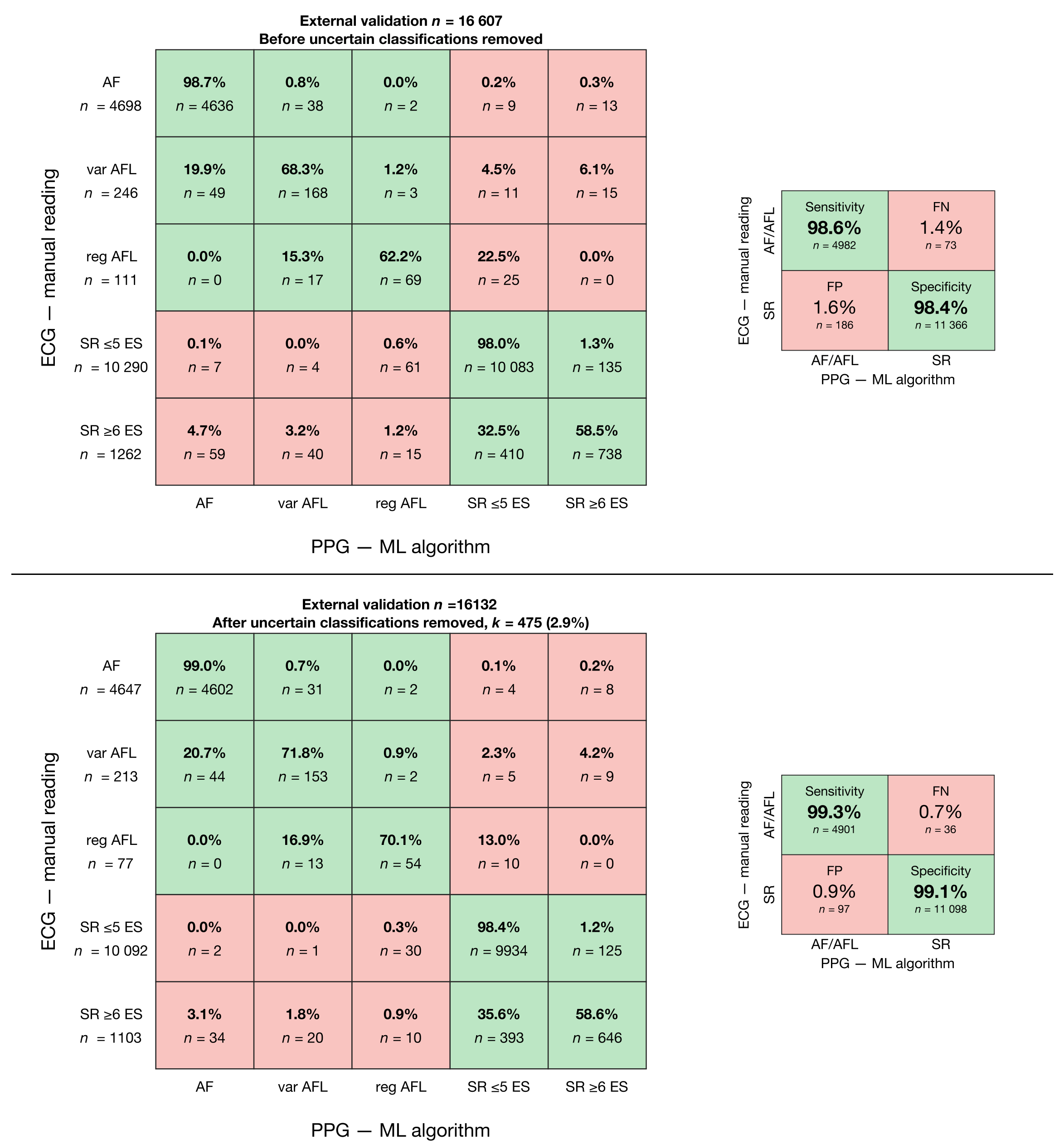

Supplementary figure S4 Confusion matrices for the external validation, including AFL recordings, before **(top)** and after **(bottom)** removal of PPG recordings with a low degree of classification certainty. In the 2x2 matrices **(right)** AF and AFL recordings are grouped together. AF: atrial fibrillation; var AFL: atrial flutter with variable AV conduction; reg AFL: atrial flutter with regular AV conduction; SR: sinus rhythm; ES: extrasystolic beats; PPG: photoplethysmogram; ECG: electrocardiogram; ML: machine learning; FN: false negatives; FP: false positives;

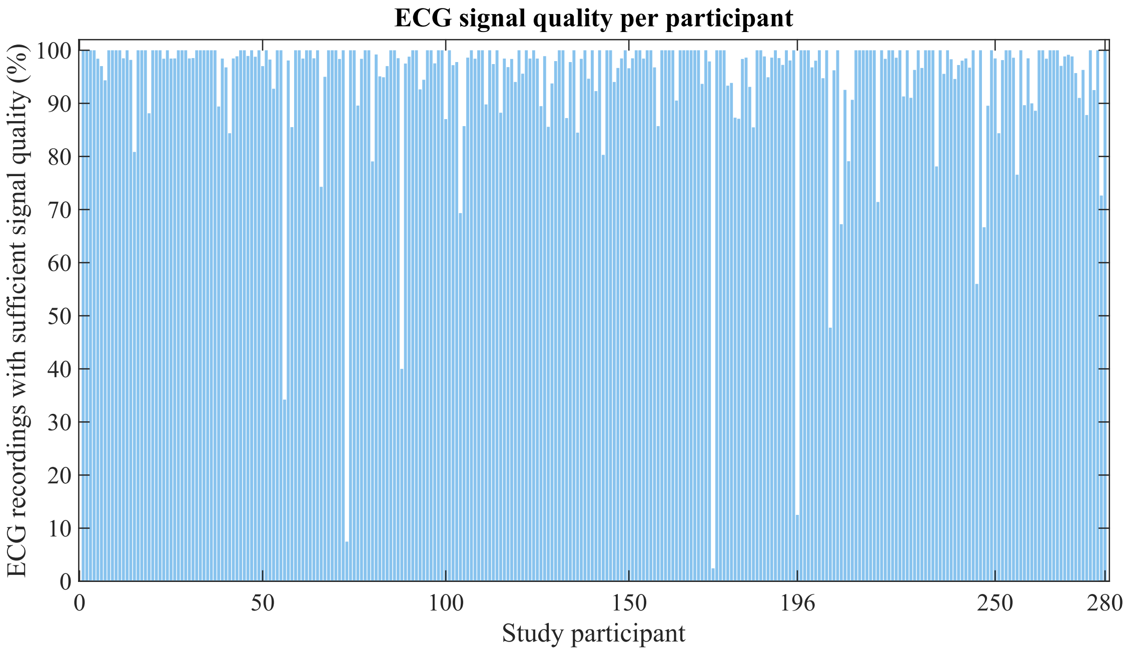

Supplementary figure S5 Signal quality of the single-lead ECG recordings (KardiaMobile) for each of the 280 study participants in the external validation cohort. The proportion of single-lead ECG recordings with sufficient quality to make a heart rhythm diagnosis, as decided by manual reading, is shown on the y-axis. The study participants received automatic real-time feedback on the user handling from the CORAI Heart Monitor application during the recordings to aid in preserving the signal quality of the single-lead ECG recordings.
ECG: electrocardiogram;

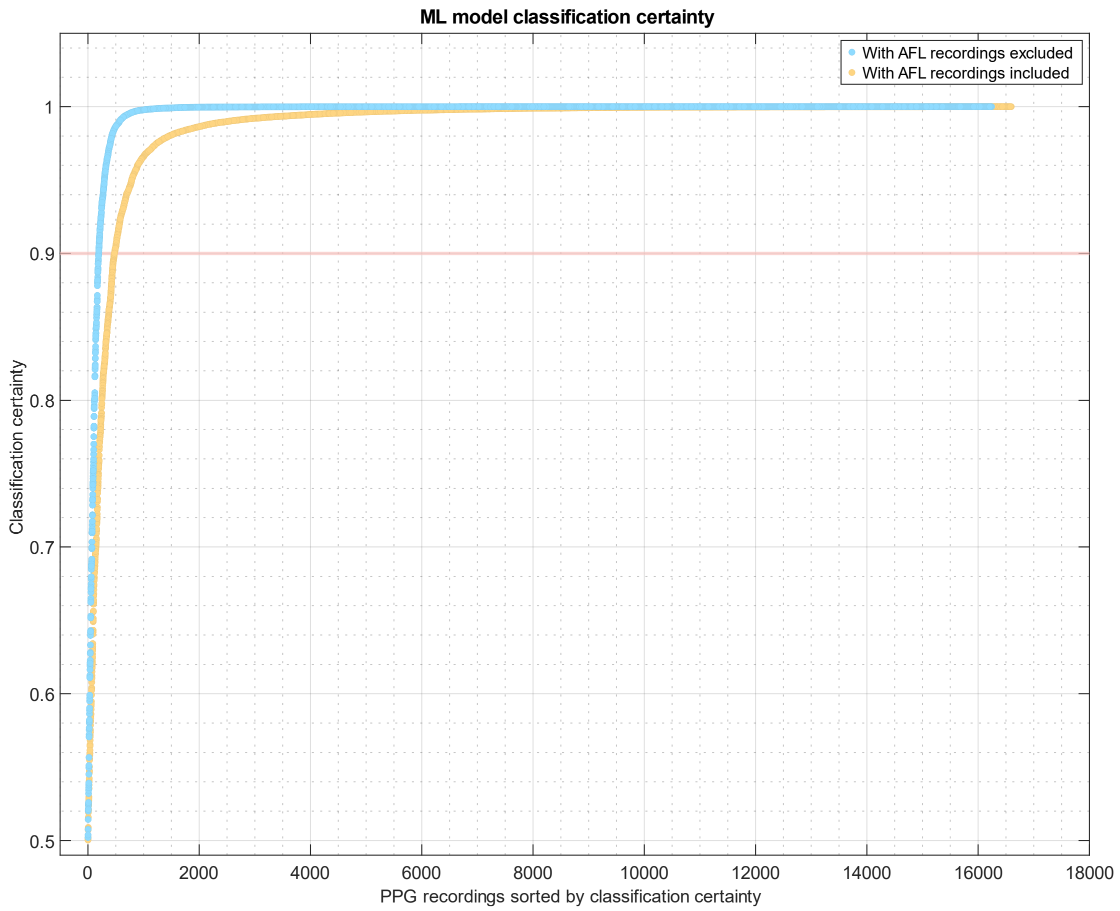

Supplementary figure S6 Sorted classification certainty for the PPG recordings in the external validation cohort for the ML classifier trained with and without AFL recordings. PPG recordings with a classification certainty < 0.9 was considered as having low certainty.
AFL: atrial flutter; PPG: photoplethysmogram; ML: machine learning;

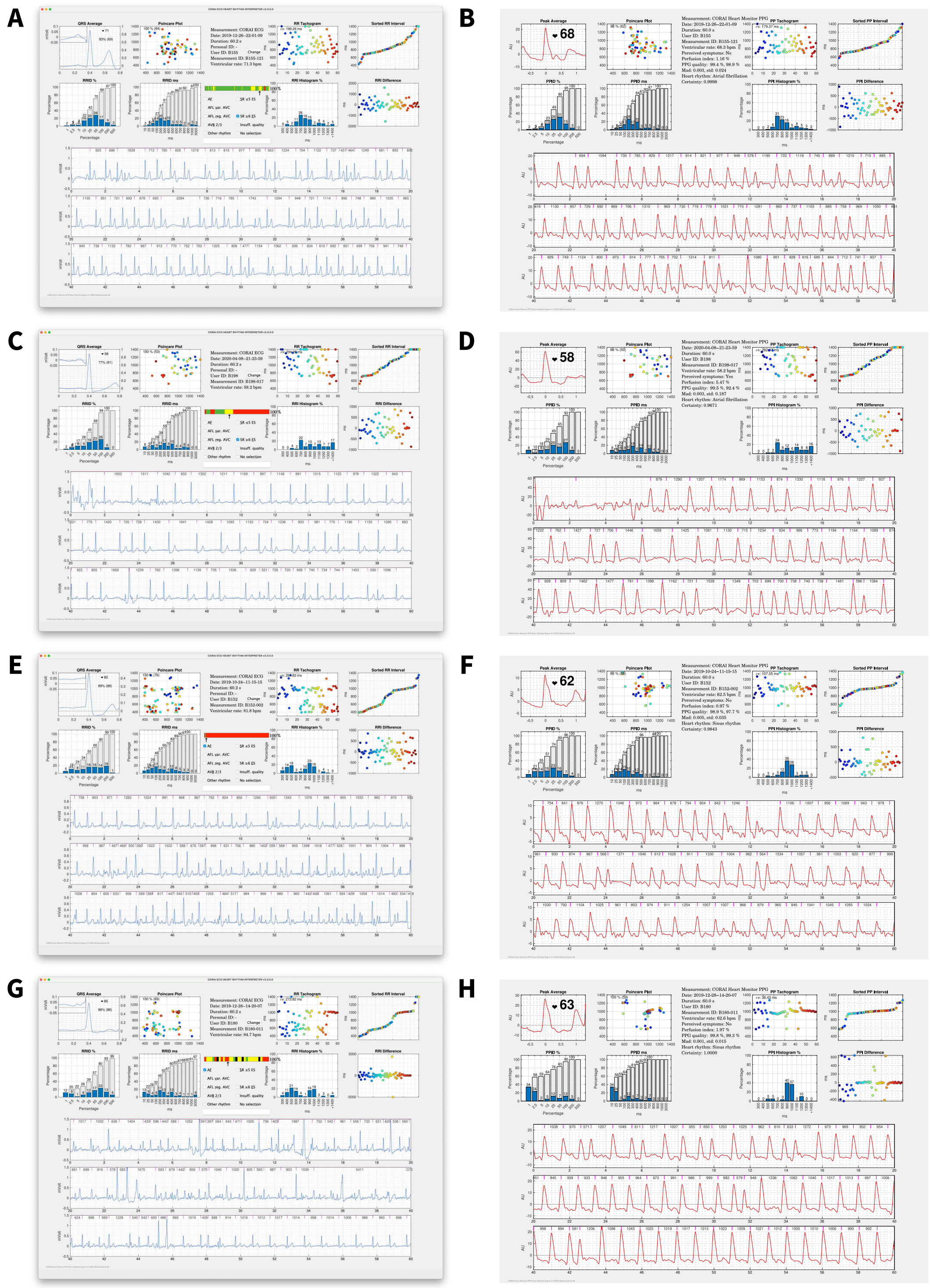

Supplementary figure S7 Examples of recordings where the heart rhythm diagnosis from manual reading of 1L-ECG **(left)** and the diagnosis of the simultaneous smartphone-PPG **(right)** from the ML-based classification algorithm did not match.
In **(A-D)** the diagnosis from ECG was SR, and from PPG it was AF (FP results).
In **(E-G)** the diagnosis from ECG was AF, and from PPG it was SR (FN results).

Supplementary data. https://doi.org/10.1093/europace/euaf031

AF: atrial fibrillation; var AFL: atrial flutter with variable AV conduction; reg AFL: atrial flutter with regular AV conduction; SR: sinus rhythm; ES: extrasystolic beats; PPG: photoplethysmogram; ECG: electrocardiogram; ML: machine learning; FN: false negatives; FP: false positives;

Supplementary table S1 The set of heart rhythm categories available to choose from during manual reading of ECG recordings in the external validation cohort.
ECG: electrocardiogram;

Atrial fibrillation	Sinus rhythm, with ≤ 5 extrasystoles
Atrial flutter, with regular AV conduction	Sinus rhythm, with ≥ 6 extrasystoles
Atrial flutter, with variable AV conduction	Insufficient signal quality
AV block II/III	Other rhythm
